# The complete mitochondrial genome sequence of *Ctenochaetus tominiensis* (Actinopteri, Acanthuridae)

**DOI:** 10.1080/23802359.2021.2016078

**Published:** 2022-01-05

**Authors:** Chen Jingni, Hu Haihao, Huang Jiantao, Yan Xiuying

**Affiliations:** Guangdong Key Laboratory of Pathogenic Biology and Epidemiology for Aquatic Economic Animals, Guangdong Ocean University, Zhanjiang, China

**Keywords:** *Ctenochaetus tominiensis*, mitochondrial genome, phylogenetic analysis

## Abstract

We sequenced and annotated the complete mitochondrial genome of *Ctenochaetus tominiensis* (Randall 1955) from Indonesia. The genome was assembled into a circular molecule of 16,429 bp with 44.45% GC content. This genome consisted of 13 protein-coding genes (PCGs), 22 tRNA genes, two rRNA genes, and 1D-loop. Phylogenetic analysis based on 13 PCGs showed that *Ctenochaetus* and *Acanthurus* were recovered in a single clade. The mitochondrial genome of *C. tominiensis* is helpful for species identification and phylogenetic position of fish.

*Ctenochaetus tominiensis* (Randall 1955) is a widely distributed fish species in the Western Pacific, Indonesia, the Philippines, New Zealand, and is mostly found around coral reef. This fish species is attributed to Acanthuridae, Acanthurinae, *Ctenochaetus*. *Ctenochaetus tominiensis* has a thick-walled stomach similar to *Acanthurus* (Clements et al. 2003). Analysis on the complete mitochondrial genome of fish will provide the basis for species identification and biological diversity (Sorenson et al. 2013).

*Ctenochaetus tominiensis* was collected from Bali, Indonesia (115°2′E, 8°7′S). A specimen was deposited at Guangdong Ocean University (Qingzhu Chu, 452714874@qq.com) under the voucher number hdfh00685. Genomic DNA was extracted with DNAzol™ Reagent from muscle tissue of three *C. tominiensis*. DNA was used to construct the library for sequencing. The mitochondrial genome was sequenced using Illumina HISeq PE150 platform (Sangon Biotech, Shanghai, China). BBMap software (https://escholarship.org/uc/item/1h3515gn) was used to process raw data. The mitochondrial genome was assembled and annotated using NOVOPlasty (Dierckxsens et al. 2017) and MITOS2 software (Gomes de Sá et al. 2017) with *A. leucosternon* EU136032 as reference. The complete mitochondrial genome of *C. tominiensis* is circular and 16,429 bp long. The base composition of this mitochondrial genome is 29.22%A, 26.33%T, 28.60%C, and 15.85%G. The mitochondrial genome contains 13 protein-coding genes (PCGs), 22 tRNA genes, two rRNA genes, and one non-coding region (D-loop).

The phylogenetic position of *C. tominiensis* was analyzed by phylogenetic trees based on 13 PCGs of 19 fishes, with *Sargocentron* as the outgroup. All sequences were aligned using DNAstar software, and evolutionary models of 13 PCGs were simulated using ModelTest3.8 software. Neighbor-joining, maximum parsimony (MP), minimum evolution, and UPGMA trees were constructed using MEGA6.0 software. MrBayes tree was constructed using MrBayes3.12 software with the general time-reversible + gamma + invariants model. The MP trees were also built using PAUP4.10 by running the heuristic search. The topological structures of all phylogenetic trees were basically similar (Figure 1). *Ctenochaetus tominiensis* with other species of *Ctenochaetus* and *Acanthurus* were recovered in a single clade, which is consistent with other studies (Sorenson et al. 2013; Grulois et al. 2020). The phylogenetic position of other basically coincided with traditional taxonomy. *Ctenochaetus* and *Acanthurus* should be synonymized when evolution studied.

**Figure 1.**
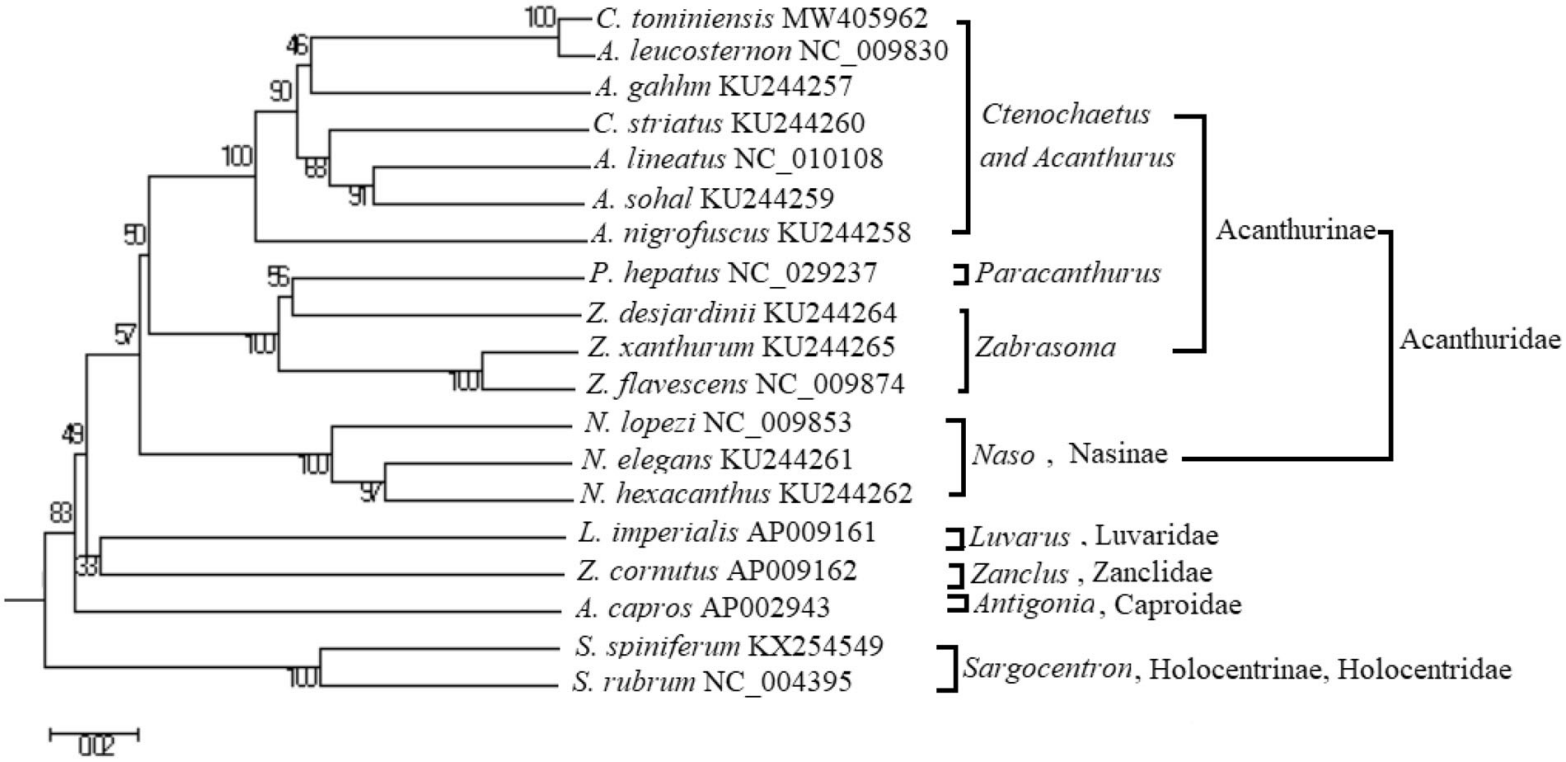
UPGMA tree based on 13 PCGs (numbers are posterior probabilities).

## Data Availability

The genome sequence data that support the findings of this study are openly available in GenBank of NCBI at https://www.ncbi.nlm.nih.gov/nuccore/MW405962 under the accession no. MW405962. The associated BioProject, Bio-Sample, and SRA numbers are PRJNA718725, SAMN18559513, and SRR14163267, respectively. Mitochondrial sequences of *Acanthurus leucosternon* (NC_009830), *Acanthurus lineatus* (NC_010108), *Acanthurus shoal* (KU244259), *Acanthurus nigrofuscus* (KU244258), *Acanthurus gahhm* (KU244257), *Ctenochaetus striatus* (KU244260), *Paracanthurus hepatus* (NC_029237), *Zebrasoma flavescens* (NC_009874), *Zebrasoma xanthurum* (KU244265), *Zebrasoma desjardinii* (KU244264), *Naso lopezi* (NC_009853), *Naso hexacanthus* (KU244262), *Naso elegans* (KU244261), *Luvarus imperialis* (AP009161), *Zanclus cornutus* (AP009162), *Antigonia capros* (AP002943), *Sargocentron rubrum* (NC_004395), and *Sargocentron spiniferum* (KX254549) are open at https://www.ncbi.nlm.nih.gov.
